# Barriers and Facilitators for the Use of a Medical Mobile App to Prevent Work-Related Risks in Pregnancy: A Qualitative Analysis

**DOI:** 10.2196/resprot.7224

**Published:** 2017-08-22

**Authors:** Adeline V Velu, Monique DM van Beukering, Frederieke G Schaafsma, Monique HW Frings-Dresen, Ben WJ Mol, Joris AM van der Post, Marjolein Kok

**Affiliations:** ^1^ Academic Medical Center Department of Obstetrics and Gynecology University of Amsterdam Amsterdam Netherlands; ^2^ University Medical Center Department of Public and Occupational Health/Amsterdam Public Health Institute Vrije Universiteit Amsterdam Netherlands; ^3^ Academic Medical Center Coronel Institute of Occupational Health University of Amsterdam Amsterdam Netherlands; ^4^ Robinson Institute University of Adelaide Adelaide Australia

**Keywords:** qualitative research, mobile app, smartphone, pregnancy, work, occupation, exposure, eHealth, mHealth

## Abstract

**Background:**

The number of women participating in the labor market in Europe has increased over the last several decades. At the same time, there is growing evidence that certain conditions of employment during pregnancy may have a negative influence on pregnancy outcomes. In order to better inform pregnant women, we aim to develop an app to help assess the health risk as a result of personal and work-related factors and provide personal advice for these women and their health care providers.

**Objective:**

The aim of this study was to compose a thematic overview of the perceived facilitators and barriers according to pregnant women, medical professionals, and employers for the use of a mobile app in obstetrical care to prevent occupational-related pregnancy complications.

**Methods:**

Two multidisciplinary focus group meetings with in total 14 participants were conducted with pregnant women, occupational physicians, general practitioners, midwives, obstetricians, and representatives of trade unions and employer organizations. Transcripts were analyzed by qualitatively coding procedures and constant comparative methods.

**Results:**

We identified 24 potential facilitators and 12 potential barriers for the use of the app in 4 categories: content of the app, the app as a mean to provide information, ease of use, and external factors. The 3 main facilitators identified were the need for a good interaction between the app and the user, apps were viewed as a more practical source of information, and the information should be understandable, according to the existing guidelines, and well-dosed. The 2 main barriers for use were extensive battery and memory use of the smartphone and sending frequent push notifications.

**Conclusions:**

The results of this study are important considerations in the developing process of a medical app implementing a guideline or evidence-based information in practice.

## Introduction

Currently the employment rate among women aged 20 to 64 years is 64% in Europe [1]. Around 57% of women in the labor force in the Netherlands are of childbearing age [2]. At the same time, there is growing evidence that certain conditions in employment during pregnancy may have a negative influence on pregnancy outcomes. For instance, working long hours in a day or working night shifts, physically demanding work, stress, and chemical, pharmaceutical, or biological exposure can potentially cause preterm birth, low birthweight, spontaneous abortion, stillbirth, and fetal abnormalities [3-9].

Pregnant women are often unaware of potential work-related risks to their pregnancy [10]. Estimations are that only 25% of employed pregnant women receive adequate counseling on work-related risks during their pregnancy [11]. Furthermore, van Beukering et al [12] concluded in a literature study that around 25% of pregnant women in the Netherlands come in contact with above mentioned work-related risks.

If pregnant women would receive more information about potential risks in their work situation, this could lead to better work adjustments. In the Netherlands, occupational physicians developed a guideline for healthy working conditions during pregnancy and the postpartum period [13]. This guideline provides clear advice on necessary adjustments to potential harmful working conditions. With these work adjustments, a healthy working environment can be created and prevent negative pregnancy outcomes in certain cases.

Mobile health (mHealth) was defined by the World Health Organization as the use of mobile devices (mobile phones, patient monitoring devices, and personal digital assistants) for medical and public health practice [14]. The benefits of mHealth interventions include that they can be delivered anywhere at any time and they provide opportunities for interactivity and tailoring to specific groups [15]. Mobile apps and smartphones are increasingly used in health care by both health care workers and the general public. In 2015, about 94% of the population in the age category of 25 to 45 years owned a smartphone with Internet access in the Netherlands [16]. Although these data were not specified by gender, it is likely that the use of smartphones is comparable between men and women in this age category. The promising research results of apps in health care, combined with the fact that smartphones are widely used by many women of childbearing age, gives smartphones the potential to further improve maternity care as an addition to the traditional health care system [17].

For pregnant women, several mHealth interventions or apps were previously developed for the care of diabetes [18-21], achieving less gestational weight gain [22-24], and support of smoking cessation [25-27]. The effectiveness of these interventions showed promising results, although most of these studies did not show significant effects on health outcomes mainly due to small sample sizes [18-23,25,27]. A recent large study in the Netherlands, however, did show significant improvement of nutrition and lifestyle due to an mHealth platform in 603 pregnant women and 1275 couples contemplating pregnancy [28].

Previous research has shown that the user satisfaction of an app or mHealth intervention is high among active users, and most are viewed as helpful, useful, and convenient [25,27,29-31]. However, continued use lagged behind and drop-out rates were high [22,32,33]. For instance, in a large nationwide email-based health promotion program for pregnant women in the Netherlands, 45% ceased participating or never opened an email. Only 16% opened all emails received and were considered very active [32]. Therefore, it is important to evaluate potential facilitators and barriers for the use of an app during the development phase to achieve good and continued use of such an app.

In order to better inform pregnant women, we wanted to develop an app to help assess health risk as a result of personal and work-related factors and provide personal advice for women and their health care providers. In doing so we wished to create awareness of work-related risks and empower pregnant women to discuss necessary work adjustments with their supervisor and potentially prevent negative pregnancy outcomes. To our knowledge, there is no literature on the use of an app that provides personal advice for pregnant women addressing work-related risks and relevant work adjustments.

This study was the first phase of a 3-phase pilot study. After this phase, the prototype of the app will be tested for usability during a think-aloud study among pregnant women. The third phase will consist of a powered study comparing the app as an addition to standard care with standard care alone. These phases are based on models for developing new tools as studied by Elwyn et al [34] and Shorten et al [35].

The aim of this study was to compose a thematic overview of the perceived facilitators and barriers by pregnant women, professionals, and employers for the use of an app in obstetrical care to reduce occupational-related pregnancy complications.

## Methods

### Overview

We performed qualitative research by conducting 2 multidisciplinary focus group meetings with a total of 14 participants. We decided to conduct multidisciplinary focus groups to involve all the stakeholders and thereby evaluate a variety of opinions of both the end-users and professionals. The methods and results were reported according to the Consolidated Criteria for Reporting Qualitative Research [36]. The ethics board of the Academic Medical Center confirmed that the Medical Research–Involved Human Subjects Act did not apply to this study.

### Participants

Participants were selected by purposive sampling of stakeholders involved in occupational health and obstetrical care and contacted by email and telephone. The inclusion criteria were that the participants were either pregnant women, occupational physicians, general practitioners, midwives, obstetricians, or representatives of trade unions and employer organizations. Participants who did not speak the Dutch language fluently were excluded. In total, we invited 30 potential candidates, between 4 and 6 from each stakeholder category, to ensure an adequate number and variety of stakeholders in both focus groups. Invitations were only declined because of previous engagements, not because of unwillingness to participate.

### Procedure

Two focus group meetings were conducted in 2015 to identify potential facilitators and barriers for the use of an app for pregnant women to prevent work-related risks during pregnancy. Prior to the meeting, confidentiality was assured and the process of the focus group was explained to the participants. All participants signed an informed consent form. Both meetings were audiotaped and fully transcribed afterwards. The focus group meetings were both facilitated by FS (female, occupational physician, senior researcher at a Dutch academic medical center, experienced in facilitating focus group). MvB (female, researcher on this project, occupational physician) and SD (female, coordinator of the regional network of birth care) took field notes. The duration of each meeting was planned for 2 hours; meetings were conducted in Dutch.

During the first part of the meeting, participants were briefly introduced to the background and aims of the project. Next, the participants were asked to respond to several questions about their knowledge and experience with pregnancy and work. We also asked about their knowledge of the Dutch guideline on pregnancy and work [13] and about experiences with health apps, mainly focused on lifestyle adjustments in general.

Subsequently, several examples of existing health apps were presented, followed by a discussion based on 5 predetermined statements (all questions, topics, and statements are shown in Multimedia Appendix 1).

### Data Analysis

The transcriptions of the focus groups were structured and analyzed with MAXQDA (VERBI GmbH), a software program to assist qualitative data analysis. For the analyses of focus group transcripts, coding procedures and the constant comparative method developed by Strauss [37] were used. Coding was divided into 3 phases starting with open coding, axial coding, and selective coding. This is a frequently used inductive, bottom-up method for analyzing qualitative data without a predetermined theoretical framework [38].

First, each of the 2 researchers (AV, MvB) started with an open coding process by examining the transcripts of the focus groups in order to assign a series of codes that were then grouped into similar concepts [39]. To ensure consistency and intercoder reliability, the 2 focus group transcripts were independently coded by the 2 researchers. Discussions between the 2 researchers resulted in a consensus list of preliminary codes. In case of discussion on the interpretation of the codes, a third researcher (FS) was involved in the process. Second, according to the axial coding process, recurrent themes within the transcripts were selected, and text fragments were sorted according to the thematic framework that appeared during the axial coding process, divided in main and subcodes. All codes were analyzed for influencing the use of the app, either in a positive way by stimulating the usage of the app coded as a facilitator or in a negative way coded as a barrier. Some citations could be interpreted as both a facilitator and a barrier. Consensus meetings between researchers led to the final categorization of themes as described in the section below.

## Results

### Overview

Each focus group consisted of 7 participants. The basic demographics of the participants are shown in Table 1. We successfully achieved the aim that in both meetings all different stakeholders were represented.

During the focus group meetings, the participants identified 24 potential facilitators and 12 potential barriers for the use of our app which were classified into 4 main themes: content of the app, the app as a means for providing information, ease of use, and external factors of influence. The barriers and facilitators in each main theme will be discussed below.

**Table 1:** Basic demographics (N=14).

Characteristics		n (%)
**Gender**		
	Male	2 (14)
	Female	12 (86)
**Occupation**		
	Midwife	2 (14)
	Obstetrician	1 (7)
	Occupational physician	3 (21)
	General practitioner	2 (14)
	Employer	2 (14)
	Labor union	1 (7)
	Physician at unemployment insurance agency	1 (7)
	Pregnant woman	2 (14)

### Theme 1: Content of the App

#### Overview

The facilitators and barriers regarding content of the app can be divided in 2 subcategories: the content of provided information and provided advice and the added value of the app compared to existing apps. The 2 categories given most value by the participants are the content of the provided information and advice. Both categories can also be subdivided into personal information and advice specified to the individual user based on her previous responses about her work situation and a more general information and advice which applies to every working pregnant woman.

#### Content of the Provided Information and Advice

Participants agreed on the fact that facilitators related to general information and advice content are keeping the advice clear and simple and to mainly indicate the urgency or importance to follow the advice instead of going into too many details and background.

All information should be easily understandable for all users, and the information and advice should be in line with the existing guideline [13].

A second strong facilitator is the ability to provide specific personal information and advice by using selective questions. This way, it is possible to determine if the user is at risk for a certain complication and synchronize the advice with the gestational age.

You wanted a start question, how did you call it, a selective question?...Do you work in one of the following occupations, you should do that.

MB, insurance physician, FG2

You should actually be able to turn off information that is irrelevant to you. I do not work with toxic agents, so everything about that is irrelevant to me...I tune out if there are, say, two pages about that.

MH, employer and pregnant woman, FG1

Informing the pregnant woman about the changes in her body and the development of the fetus will improve her understanding of the effects the pregnancy may have on her work situation and vice versa. Possible adverse outcomes of the pregnancy are also important to mention in the app. Women with high-risk pregnancies could particularly benefit from specific and more personalized advice for their situation.

A potential barrier is the risk of users interpreting the information themselves without seeking further professional advice. One participant pointed out that a risk profile based on a questionnaire in the app cannot be compared with an actual conversation between a physician and a pregnant woman because the app only works with the input of the user herself. This makes the reliability of personal advice difficult to interpret and could become a barrier related to the content of the app.

Refer really fast to a gynecologist or midwife or indeed the occupational physician. Otherwise you will indeed risk that the pregnant woman herself will interpret medical information or interpret risk factors.

FM, occuptational physician, FG 1

#### Added Value of the App Compared to Existing Apps

Participants considered it a facilitator if the new app had added value with respect to other existing apps. Examples mentioned in the focus groups to create added value were (1) develop an app based on medical knowledge and guidelines, (2) cover the preconception and postpartum periods in addition to the pregnancy period, and (3) make the app noncommercial.

### Theme 2: App as a Means for Providing Information

In this category, the focus groups reported mainly facilitators in relation to the app, most importantly the practical aspects. Moreover, apps were viewed as faster and easier in searching for information and the information was available at every place and every time.

Always at hand. Since that is the power of an app. You always have it on you. You can consult it anytime.

AR, labor union, FG 1

The fact that pregnant women already receive a large amount of information regarding their pregnancy can be interpreted both as a facilitator and as a barrier for the app. One point of view, as reported by the participants, is that the app is more easily accessible than printed information and therefore a facilitator for use. On the other hand, a few participants mentioned that the app provides more information and there is already enough information available.

### Theme 3: Ease of Use

#### Overview

The facilitators and barriers for the ease of use of an app can be divided into 3 subcategories: technical aspects of the app, feedback and interaction between app and user, and reaching the target users by the mode of delivering the information to the user.

#### Technical Aspects

Participants described as facilitators the fact that games and quizzes make an app more fun to use. Another important facilitator for the use of the app was that it is only applicable for a set period of time and you can delete it after 9 months because apps that are not used frequently will be deleted, according to our participants.

Potential barriers that should be kept in mind are that there are numerous existing operating systems, apps that use a lot of battery and memory are unpopular, and the information provided should be readable on a smartphone.

What kind of apps do you delete?

FS, facilitator, FG 2

[Apps that use] lots of memory, lots of power. Apps that are very active, in that case your battery goes down...

HB, general practitioner

#### Feedback and Interaction Between App and User

Overall consensus was that interaction between the user and the app strongly stimulates the use of an app. But the opinions on interaction also showed some inconsistencies between participants, and sometimes in participants their opinions seemed to vary. Several participants emphasized that messages about the development of the fetus and changes in the female pregnant body are informative and entertaining and facilitate the use of the app. Also, reminders of specific personalized advice based on an earlier risk analysis in the app were evaluated as helpful and welcome.

On the other hand, every participant criticized push notifications defined as frequent uncalled-for messages. One participant also mentioned that these push notifications can be risky when users have an adverse pregnancy outcome. Suggested solutions to this issue were to offer the option to sign out of the app in case of adverse pregnancy outcome or only show new general notifications when the user opens the app itself.

I fully recognize that. Because I do not have an app, but I do receive emails from an organization. And then you see the changes in your body and of the baby week by week, and say, and those of the baby. So in that respect I think receiving it through an app is useful. So you see the growth, and like, we are now in week 34; this is happening with your child. And you should adjust your health to your work et cetera. So I believe that would be very good.

DD, pregnant woman, FG 1

But do you delete apps that for instance send very many push notifications?

FS, facilitator, FG 1

Yes, I always turn them off immediately.

CvW, employer

Those are very irritating.

MP, gynecologist

Yes, those are very irritating.

AR, labor union

#### Reaching Target Users by the Mode of Delivering the Information

Three main facilitators were identified related to the mode of delivering information: the content of the app must be understandable, the information should be well-ordered, and the information should be supplemented with illustrations, video fragments, and icons to improve clarification.

If you reduce the text and do not use extensive amounts of text and work with icons that already helps.

CdG, pregnant woman, FG 2

The comprehensiveness of the app was considered an important facilitator and as such subject of long debate. Several suggestions were given to achieve a comprehensive app on pregnancy and work. For instance, participants felt that offering the app in multiple languages (Dutch, English, Spanish, and Polish were named as important; Moroccan and Turkish were questioned if they were still necessary), using plain language, and having a text-to-speech function can improve accessibility of the app for all users.

Providing too much information was viewed as a barrier by the participants, risking less usage of the app. Options to avoid this barrier could be to create a hyperlink in the app for further information and give users the option to read more if desired. On the other hand, the app should not be needlessly complicated with too many hyperlinks.

### Theme 4: External Factors

#### Obstetrical Caregivers

Obstetrical caregivers such as gynecologists and midwives are facilitators by supporting the app, according to the participants. They work according to the occupational physician practice guideline [13] and believe in screening for work-related risks as part of the standard care. For it to become standard care, this knowledge should be implemented in the education for midwives. A second option could be to actively involve the obstetrical caregivers or create an extra app for the caregivers to use.

#### Employer, Supervisor, or Company

The employers can potentially be very strong facilitators for the use of the app. Unfortunately the participants in the focus groups mainly identified barriers for the app. The participants thought that employers may have a negative prejudice about work adjustments for pregnant women. Work adjustments can be seen as more bothersome than sick leave, and the entire organization, including colleagues, might not understand fully the need for adjustments. Employers have little knowledge about work-related risk factors for pregnant women, and many may not see that it is in their own best interest to implement well-timed work adjustment that could lower the risk of sick leave. Therefore, they may not stimulate the use of the app. Participants also pointed out that the app might cause a disturbance in the relationship between a pregnant woman and her employer. To prevent occupational conflicts, the advice in the app should be formulated cautiously and should emphasize stimulating a constructive dialogue.

Yes, I have experienced that myself, so to speak. That I basically did not dare to step up to my employer, when the last 2 weeks were quite heavy. I was aware that I was entitled to extra breaks, et cetera, but somehow I was afraid to speak up. So I do understand the story you just told, that when an employee shows up with solely an app, and the employer is not informed that this situation might give some, well, disruption, so to speak.

CvW, employer, FG 1

A significant factor in preventing these barriers is informing and involving the employers and organizations. If employers see the usefulness of the app itself and the importance of sustainable work during pregnancy, they may become more involved as facilitators for the use of the app by their employees.

The fact that there is a large variety in type and size of employers and companies is neither a facilitator nor a barrier for the use of the app. A footnote was placed by some participants that the app should be developed irrespective of the willingness of employers to participate. It cannot be expected that an app will change the entire culture of companies.

#### Government

One participant suggested that a television commercial from the ministry of health might facilitate the use of the app.

**Table 2:** Development of a mobile app: thematic overview of facilitators and barriers.

Theme and subcategory		Facilitators	Barriers
**Theme 1: Content of the app**			
	**Content of the provided information and advice**		
		Understandable information (general)	
		Information and advice according to the existing guidelines (general)	
		Keeping advice clear and simple (general)	
		Showing only relevant and personal information to the user (personal)	Reliable personal advice is difficult when the risk profile is based only on a questionnaire (personal)
		Providing information on the changes in the pregnant body and development of the baby to better understand the impact on her work situation (personal)	It’s important to provide some general advice to every user; the app shouldn’t become too personal (personal)
		Using a selective question to determine if the user is at risk for a certain risk factor (personal)	
		Synchronizing the advice with the gestational age (personal)	
		Providing specific advice in case of adverse pregnancy outcome (personal)	
	**Added value compared to existing mobile apps**		
		App should be based on medical knowledge and the guideline	
		Cover the preconception and postpartum periods in addition to the pregnancy period	
		Make the app noncommercial	
**Theme 2: App as a means for providing information**			
	**Practical aspects**		
		App is easier and faster for searching for information and is always available	
	**Pregnant women already receive a lot of information regarding their pregnancy**		
		App is more accessible than printed information	App provides even more information when there is already enough
**Theme 3: Ease of use**			
	**Technical aspects**		
		Games and quizzes make the app more fun to use	There are several different operating system
		The app is only useful for 9 months and can be deleted afterwards	Creating an app that uses a lot of battery and memory of the smartphone
			Information should be readable in a smartphone; no pdf documents
	**Feedback and interaction between app and user**		
		Providing messages about the development of the fetus and the pregnant body	Push notifications are unwanted
		Providing reminder messages of specific and personal advice based on earlier risk analyses	Push notifications are risky in case of adverse pregnancy outcomes
	**Reaching the target users by mode of delivering the information**		
		Content of the app should be understandable for every user	Providing too much information
		Information should be well-ordered	
		Illustration, icons, and videos can provide clarification	
		Offer the possibility of linking to more information if desired	
**Theme 4: External factors**			
	**Obstetrical caregivers**		
		Obstetrical caregivers support the app	
	**Employer, supervisor, or company**		
		Employers are important for the app to succeed	Employers have little knowledge about work-related risk factors for pregnant women and don’t see the benefit for themselves
			Employers might have a negative prejudice about work adjustments for pregnant women
			App can cause disturbance
	**Government**			
		A television commercial might stimulate the app	

## Discussion

### Principal Findings

In this study we aimed to compose a thematic overview of the perceived facilitators and barriers for pregnant women, professionals, and employers for the use of a mobile app in obstetrical care to prevent occupational-related pregnancy complications.

We identified 24 facilitators and 12 barriers within 4 categorical themes, of which we identified 3 main facilitators and 2 main barriers to the successful implementation of our app in obstetrical care to reduce occupational-related pregnancy complications. The most important facilitator, in the opinion of our participants, is the need for good interaction between the app and the user to make the app personal to the user. The second facilitator is the fact that apps are viewed as a more practical source of information compared to traditional printed information. The third main facilitator is that the information should be understandable, according to the existing guidelines, and well-dosed. Additional information should be hyperlinked.

As barriers, several technical aspects may have negative influences on the use of the app according to our participants. Extensive battery and memory use of the smartphone are considered barriers. The second important barrier mentioned by the participants was sending frequent push notifications.

### Comparison With Prior Work

Previous qualitative health studies on mHealth and eHealth in obstetrical health care mainly investigated (personalized) text messages [27,40-42] or Internet-based programs [35,43]. Most of our findings are comparable to these studies, especially the interactive and personalized aspects; our participants emphasize that a personalized tool which provides only relevant and specific information for the user is a very strong facilitator for the use of the tool [35,40-43]. Tripp et al [17] also showed that apps with interaction between the app and the user were shown to be the most popular kind of apps in obstetrical care. Furthermore, findings from the qualitative research of Naughton et al [27] on attitudes toward text message smoking cessation support suggests that maximizing personalization and personal relevance can increase the value of text message support and reduce the risk of disengagement.

Since the main purpose of our app is to provide detailed information and advice on work adjustments in certain specific work-related risks in pregnancy, the personal and interactive aspects of the app could be of strong positive influence for our app.

The fact that apps are viewed as faster and easier in searching for information and the information is always available at every place and every time has been pointed out in previous research as well [41,44].

Feedback from the app to the user is a complex outcome in our results since it can potentially be a strong facilitator; however, there exists a delicate balance between important stimulating reminders of advice and frequent uncalled-for push notifications, which can be experienced as a barrier. This delicate balance has also been recognized in previous studies. Two studies reported that text messages could stimulate positive behavioral changes [40,42], and one study reported that even more frequent messages would be appreciated [30]. On the other hand, our participants expressed frequent messages as a point of concern. These concerns are in line with the results in the study by Dennison et al [44]. Reminders of advice are well accepted and considered useful, which is also supported by other studies [30,40,42,44]. The mixed method qualitative study of Knight-Agarwall [30] showed participants using an app to monitor gestational weight gain wanted pop-up messages as a reminder to undertake certain activities [30].

### Strengths and Limitations

A strength of our study is the proper qualitative health method we used for the focus group meetings and the analysis of the data. Furthermore, both focus group meetings were facilitated by the same experienced facilitator. Both meetings were audiotaped and fully transcribed and were independently coded by 2 researchers, resulting in negligible intercoder variance. In case of discussion on the interpretation of the codes, a third researcher was involved in the process.

Other strengths of our study are that in both focus group meetings all different stakeholders were represented, which created the aimed interaction between stakeholders. Such group dynamic and diversity stimulated a broad view on the topics discussed and did not prevent reaching consensus on important issues [45].

In line with previous literature on qualitative health research, our number of participants is considered sufficient [46]. Besides the sufficient number of participants, the results in both meetings were comparable and we therefore believe we have achieved data saturation.

A disadvantage of conducting focus group meetings with different stakeholders together may be reluctance to be completely honest because of possible hierarchy between the different participants. Therefore, this method might lead to potential loss of important information. The fact that in our study we chose to mix professionals and pregnant women could be considered a limitation to our study. Since the discussed subject in our study, the development of an app, is not a delicate matter and is in the best interest of all the participants, we decided that this risk was small and therefore acceptable. The active participation of all participants during the meetings also indicated no reluctance of participants to share their opinions and experiences.

### Conclusion

We have identified clear facilitators and barriers for the use of an app in obstetrical care. The correct content and dosage of interaction with the end user is a complex aspect to consider in the development of an app. These outcomes will contribute to the further developmental phases of an app. The results of this study are especially of interest to medical professionals in several areas who aim to develop an app implementing a guideline or evidence-based information in practice.

In future research we aim to evaluate the usability of the app in a think-aloud study among pregnant women. Subsequently we aim to evaluate the effectiveness of the app in a controlled trial.

## Appendix

Multimedia Appendix 1 Topics and statements of the focus group meetings.
