# Supplementary material for: Barriers and Facilitators for the Use of a Medical Mobile App to Prevent Work-Related Risks in Pregnancy: A Qualitative Analysis
Source: JMIR Res Protoc. 2017 Aug 22;6(8):e163. doi: 10.2196/resprot.7224 (PMC5585593; doi:10.2196/resprot.7224)
Supplement: Multimedia Appendix 1 [file resprot_v6i8e163_app1.pdf]

Appendix 1 – Topics and statements of the focus group meetings

Part one: Introduction.
Use of smartphone and apps. Introduction of the objective of our app.

- What is your experience with the topic Pregnancy and Work?
- What are the bottlenecks you experience?
- Are you familiar with the guideline 'pregnancy, postpartum period and work'? Do you work by the guideline regularly?
- Would you appreciate support for using the guideline?
- Do you use a smartphone for health apps? What are your experiences with the health apps?
- Which apps do you use more frequent? When do you follow the provided advice and when do you not follow the advice?
- Which apps do you delete and why do you delete them?
- Which factors are of influence for pregnant users to follow the provided advice in our app?
- What are important aspects which stimulate the use of the app?
- What could potentially work as a disadvantage and frustrate the pregnant user for the use of the app?

Part two: Discussion on the basis of statements after a short presentation with examples of existing apps.*

- Topic 1: The app as a tool to reach pregnant women:
  Statement 1: The app is the designated mean to reach all employed pregnant women. When employers and occupational health professionals do not provide the correct information we will have to provide it with an app.

- Topic 2: Content of the app and the advices:
  Statement 2: Providing a lot information or a lot of options will lead to less usage of the app. Details of the influence of for instance all the different toxins and infectious diseases do not belong in this app.

- Topic 3: Effect of the app on adjustments of the work situation:
  Statement 3: When a pregnant shop assistant receives the advice from the app to not stand more than three hours during her work, because it could be harmful for her child, it is very likely that she will arrange work changes with her supervisor.

- Topic 4: The app and health care professionals:
  Statement 4.1: Healthcare professionals in the primary care like midwifes, general practitioners and maternity nurses should stimulate the use of the app. How would that work in your opinion?

  Statement 4.2: Healthcare professionals in the secondary care like obstetricians should stimulate the use of the app. How would that work in your opinion?

- Topic 5: The app and the working environment:
  Statement 5: The working environment (employer, supervisor, human resource advisors, occupational healthcare professionals) should stimulate the use of the app. How could that be done?

- Topic 6: Reinforcing:
  Statement 6: Additionally to the provided advice the app should send encouraging messages that emphasize that the pregnant user is doing well by discussing the advice with her supervisor or occupational healthcare professional.

* The introduced excisting apps were:

- App Werk en zwangerschap FNV (Pregnancy and work, of a trade union) https://www.fnv.nl/themas/zwanger-en-ouderschap/app-werk-en-zwangerschap/
- App versterk je Enkel (Veiligheid.nl) (Strengthen your ankle (safety.nl) https://www.veiligheid.nl/sportblessures/producten-en-diensten/apps/enkel-app
- App Owise about breastcancer http://www.owise.nl/
- One sheet with pictures of random apps about food, weight loss and exercises.
